# Cross-reaction between piroxican and thimerosal diagnosed by photo patch test

**DOI:** 10.1186/1939-4551-8-S1-A159

**Published:** 2015-04-08

**Authors:** Kelly Yoshimi Kanamori, Carolina Tavares De Alcântara, Nathalia Siqueira Robert De Castro, Marcelo Vivolo Aun, Jorge Kalil, Antonio Abílio Motta

**Affiliations:** 1 University of São Paulo, Brazil

## Background

Nonsteroidal antiinflammatory drugs (NSAIDs) and antibiotics are widely used drugs and are the leading causes of adverse drug reaction (ADR), especially skin reactions. The photosensitizing effects of piroxicam are known for a long time and triggered by a photoproduct structurally similar to antigenic thiosalicylic acid present in the chemical formula of thimerosal and piroxicam. The thimerosal is a preservative used in topical products such as: antiseptics, eye drops, solutions for contact lens and vaccines. Patients sensitized to thimerosal may have photosensitivity reactions with the use of piroxicam by cross reaction to thiosalicylic acid.

## Methods

Literature review and case description.

## Results

Female patient, 37 years, from São Paulo came to our clinic for evaluation due to lombocitalgy therapy caused by a disc herniation. She reported a history of flushing, reddish plaques and itching in the body 24 hours after using diclofenac and piroxicam simultaneouly. At the Emergency Department received promethazine, methylprednisolone and dexchlorpheniramine and the lesions subsided in few hours. Another episode occurred after a hysterectomy surgery, when she received tenoxicam, dipyrone, metoclopramide and cefazolin. Next day, there were "urticaria" plaques in cervical and abdominal region. The patient was treated with antihistamine and improvement after 1 week. We performed a Patch Test with piroxicam, tenoxicam and thimerosal in the concentration of 10%. The first reading was done in 48 hours and evidenced erythema and bullous lesions for thimerosal and erythema for piroxican. The 96 hours reading showed microvesicles, erythema and pseudopods for piroxicam and erythema and microvesicles for thimerosal. The patch test was negative with tenoxicam.

## Conclusions

Piroxicam may induce photoallergy through a photoproduct, wich is structurally similar to the antigenic thiosalicylic acid present in the formula of thimerosal. There is no evidence of cross-reaction between piroxicam and tenoxicam, because this photoproduct is not formed upon irradiation of tenoxicam. The diagnosis of drug photosensitivity reaction is performed by clinical history associated with photo patch test.

## Consent

Written informed consent was obtained from the patient for publication of this abstract and any accompanying images. A copy of the written consent is available for review by the Editor of this journal.

